# Targeted genomic capture and massively parallel sequencing to identify novel variants causing Chinese hereditary hearing loss

**DOI:** 10.1186/s12967-014-0311-1

**Published:** 2014-11-12

**Authors:** Qinjun Wei, Hongmei Zhu, Xuli Qian, Zhibin Chen, Jun Yao, Yajie Lu, Xin Cao, Guangqian Xing

**Affiliations:** Department of Biotechnology, School of Basic Medical Science, Nanjing Medical University, Nanjing, 210029 PR China; Department of Otolaryngology, The First Affiliated Hospital of Nanjing Medical University, Nanjing, 210029 PR China

**Keywords:** Targeted genomic capture, Exome sequencing, Hearing loss, Gene mutation

## Abstract

**Background:**

Hereditary hearing loss is genetically heterogeneous, and hundreds of mutations in than 60 genes are involved in this disease. Therefore, it is difficult to identify the causative gene mutations involved. In this study, we combined targeted genomic capture and massively parallel sequencing (MPS) to address this issue.

**Methods:**

Using targeted genomic capture and MPS, 104 genes and three microRNA regions were selected and simultaneously sequenced in 23 unrelated probands of Chinese families with nonsyndromic hearing loss. The results were validated by Sanger sequencing for all available members of the probands’ families. To analyze the possible pathogenic functional effects of the variants, three types of prediction programs (Mutation Taster, PROVEAN and SIFT) were used. A total of 195 healthy Chinese Han individuals were compared as controls to verify the novel causative mutations.

**Results:**

Of the 23 probands, six had mutations in DFNA genes [*WFS1* (n = 2), *COCH*, *ACTG1*, *TMC1*, and *POU4F3*] known to cause autosomal dominant nonsyndromic hearing loss. These included one novel in-frame indel mutation, three novel missense mutations and two reported missense mutations. Furthermore, one proband from a family with recessive DFNB carried two monoallelic mutations in the *GJB2* and *USH2A* genes. All of these mutations co-segregated with the hearing loss phenotype in 36 affected individuals from 7 families and were predicted to be pathogenic.

**Conclusions:**

Mutations in uncommon deafness genes contribute to a portion of nonsyndromic deafness cases. In the future, critical gene mutations may be accurately and quickly identified in families with hereditary hearing loss by targeted genomic capture and MPS.

**Electronic supplementary material:**

The online version of this article (doi:10.1186/s12967-014-0311-1) contains supplementary material, which is available to authorized users.

## Introduction

Hearing loss is an extremely common problem worldwide, and it is one of the most genetically heterogeneous disorders occurring in humans. Approximately half of the cases have a genetic etiology, including nonsyndromic hearing loss (NSHL) and syndromic hearing loss (SHL). NSHL as the sole defect accounts for seventy percent or more of deafness cases, and different modes of inheritance have been observed. To date, 65 genes with more than 1000 discrete deafness-causing mutations have been identified [1]. SHL comprises the remaining 30% of hearing loss cases, and it appears accompanied by other medical or physical findings. Similar to NSHL, SHL is associated with a growing list of causative genes and hearing loss syndromes [2]. Thus, the discovery of a causative gene (mutation) of hereditary hearing loss is necessary to resolve the clinical and genetic heterogeneity of deafness.

Previously, most deafness genes (mutations) have been identified through traditional positional cloning (Sanger sequencing), which is an expensive and time-consuming process. More recently, next-generation sequencing, which is also known as massively parallel sequencing (MPS), has been introduced as an alternative approach to more traditional methods [3-5]. Whole-exome sequencing (WES) allows for the targeted enrichment and resequencing of nearly all exons of protein-coding genes and identifies genetic variation at a single base-pair resolution. Accordingly, WES uses next-generation technologies to provide a transformational approach for identifying causative mutations of Mendelian disorders. Different targeted genomic capture methods and MPS have been successfully applied to detect gene mutations in relatively small sets of deafness families [6-9]. In the present study, we performed targeted genomic capture and MPS to screen 104 genes and three microRNA regions that are known to be responsible for hereditary hearing loss in 23 unrelated probands of Chinese families with NSHL. We identified six causative variants in DFNA genes, including four novel mutations, and a novel combination of two monoallelic mutations in *GJB2* and *USH2A*. This study provides a reliable strategy for the routine genetic diagnosis of hearing loss.

## Materials and methods

### Subjects and pre-exclusion of frequently reported deafness genes

A total of 143 available members of twenty-three Chinese families were recruited from the Otology Clinic of the First Affiliated Hospital at the Nanjing Medical University, Nanjing, China. Each family had at least 2 hearing-impaired individuals. All affected members in these families were diagnosed as having hereditary NSHL by a complete hearing evaluation, general examination and medical history collection. Peripheral blood samples were obtained from available members of these families, and genomic DNA was extracted using a blood genomic DNA extraction kit (TianGen, Beijing, China) according to the manufacturer’s protocol.

Each proband of the 23 families was pre-tested for nine hotspot mutations of deafness genes that have been reported in Chinese individuals with a Deafness Gene Mutation Detection Array Kit (Capital Bio Corporation, Beijing, China), as previously described [10]. The mutations included c.35delG, c.176del16bp, c.235delC and c.299delAT in the *GJB2* gene, c.538C > T in the *GJB3* gene, c.IVS7-2A > G and c.2168A > G in the *SLC26A4* gene, and m.1555A > G and m.1494C > T in the *MT-RNR1* gene. The analysis revealed that 22 probands were negative for these mutations, and one carried the monoallelic *GJB2* c.235delC mutation. To further search for the causative genes in these families, we performed targeted genomic capture and MPS. For cases in which novel variants were detected, segregation analysis was performed to assess the family. A total of 195 ethnicity-matched individuals were selected as controls to confirm the candidate mutations. This study was approved by the Ethical Committee of Nanjing Medical University for Human Studies. All participants provided written informed consent that complied with all Declaration of Helsinki tenets.

### Targeted genomic capture and next-generation sequencing

Whole-exon regions of 104 deafness genes and three microRNA regions (Additional file 1: Table S1) were target-enriched using a Target Enrichment Kit (MyGenostics Inc., Beijing, China) as previously described [11]. A minimum of 3 μg of DNA was used to generate indexed Illumina libraries according to the manufacturer’s protocol. The final library size was 300 to 400 bp, including the adapter sequences. The enrichment libraries were sequenced on an Illumina HiSeq 2000 sequencer to generate 100 bp paired-end reads. Next, high-quality reads were identified by filtering out low-quality reads and adaptor sequences using Solexa QA package [12] and Cutadapt program, respectively. Variants were first selected if they appeared in the 1000 Genomes Project database with an MAF of >0.05, and then they were selected if they appeared in the 300 local Asian Genome database. The remaining variants were further processed according to the dbSNP database. SNPs and indels were identified using SOAPsnp and GATK programs. Subsequently, the reads were realigned to the reference genome (NCBI37/hg19) using BWA software. Non-synonymous variants were evaluated by four algorithms, including PolyPhen, SIFT, PANTHER and Pmut, to determine pathogenicity [13].

### Mutation validation and analysis

Sanger sequencing was performed on 51 available members of seven families. Seven pairs of primers surrounding the suspected variants were designed with Primer Premier 5.0 (Premier Biosoft) (Additional file 2: Table S2). PCR products were purified and sequenced with a BigDye Terminator v3.1 Cycle Sequencing Kit (Applied Biosystems, Foster City, CA, USA) and ABI 3730 DNA Sequencer (Applied Biosystems, Foster City, CA, USA) with Chromas Lite v2.01 (Technelysium Pty Ltd., Tewantin, QLD, Australia) software. DNA sequence variation was identified through a comparison of subjects’ DNA sequences to those of *WFS1* (NM_001145853), *COCH* (NM_001135058), *ACTG1* (NM_001199954), *TMC1* (NM_138691), *POU4F3* (NM_002700), and *USH2A* (NM_206933).

Possible pathogenic effects caused by the rare or novel non-synonymous SNPs were evaluated by Mutation Taster (http://www.mutationtaster.org), PROVEAN (http://provean.jcvi.org) and SIFT (with cut-off scores of −1.3 and 0.05, respectively; http://sift.jcvi.org). Multiple sequence alignments were performed using ClustalW, with the default setting of nine species.

## Results

### Deaf panel enrichment and targeted sequencing

The enriched libraries were labeled with a unique barcoding sequence, pooled, and sequenced on an Illumina HiSeq 2000 to generate paired-end reads of 100 bp in length. As shown in Table 1, the average sequencing depths of the targeted regions were 188 to 264. Over 98% coverage of the targeted regions was achieved for each proband. The coverage of the targeted exons for the >10× reads ranged from 94.10% to 96.20% and from 92.60% to 94.80% for the >20× reads. All mitochondrial DNA and microRNA regions were sequenced at a depth of over 100×. Using SOAPsnp, we identified an average of 485 to 656 variants for each sample. To give priority to identification of the deleterious mutations, we adopted a series of filtering strategies to focus on non-synonymous variants (missense, nonsense and splice variants) using a combination of filtering against HapMap 28, SNP databases and mutiple algorithms (PolyPhen, SIFT, PANTHER and Pmut). We found six deleterious non-synonymous mutations: *COCH*, p.G38D; *WFS1*, p.R653C; *ACTG1*, p.E316K; *TMC1*, p.D572N; *POU4F3*, p.P164R and *USH2A*, p.P1684L. Of the indels, a total of 5 ~ 26 coding indels initially discovered in the probands using the GATK program, only one deletion (*WFS1*, p.E680del) to be identified based on the sample filtering strategy. Through the above analysis, we successfully validated the deleterious candidate variants in the seven families.

**Table 1:** **Coverage and sequencing statistics of 7 probands**

**Family ID**	**Proband no.**	**Initial bases on target**	**Base covered on target**	**Coverage of target region (%)**	**Mean depth on (×)**	**Fraction of target covered ≥4× (%)**	**Fraction of target covered ≥10× (%)**	**Fraction of target covered ≥20× (%)**
JSNY-021	III3	335538	329163	98.10	190	96.69	94.10	92.60
JSNY-027	III4	335538	329834	98.30	188	97.00	95.80	93.50
JSNY-033	II3	335538	332518	99.10	264	97.56	95.30	93.60
JSNY-043	III3	335538	333189	99.30	259	98.00	96.10	94.80
JSNY-045	II3	335538	330675	98.55	223	97.39	95.70	93.30
JSNY-053	III4	335538	331089	98.67	199	98.19	96.20	94.50
JSNY-056	III1	335538	329965	98.34	245	96.96	94.50	92.30

### Verification of candidate mutations in seven families

**Figure 1:**
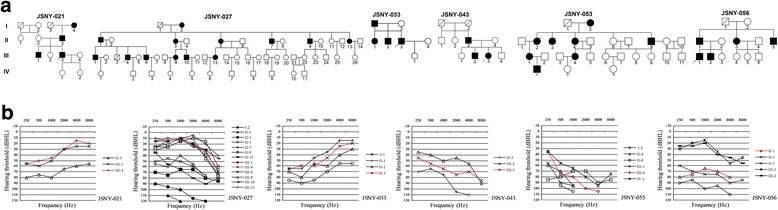
**Pedigrees and audiograms of 6 DFNA families. (a)** Family pedigrees showing autosomal dominant inheritance. Open symbols, unaffected; solid black symbols, affected; squares, men; circles, female; arrows at lower left, probands. **(b)** Pure-tone audiograms of affected members in each family. All hearing thresholds shown here are from the better ear. The red line indicates the proband.

Six families with candidate variants (JSNY-021, JSNY-027, JSNY-033, JSNY-043, JSNY-053 and JSNY-056) showed autosomal dominant inheritance (Figure 1a). For all affected members of each family, late-onset, progressive hearing loss was observed. Pure-tone audiograms showed bilaterally symmetric, sensorineural, mild-to-profound deafness (Figure 1b). With regard to audiometric configurations, two pedigrees were up-sloping (JSNY-021 and JSNY-033), and the others exhibited down-sloping or flat patterns. Aside from their hearing loss, the patients were phenotypically normal. None of them complained of vestibular symptoms. In each of the probands, multiple potentially functional variants with predicted damaging effects were identified by our approach and validated by Sanger sequencing. Each validated variant was tested for complete co-segregation with hearing loss in the proband’s family, and this assessment included an additional 28 affected individuals and 13 unaffected family members. Finally, six variants were successfully confirmed in the *WFS1* (n = 2), *COCH*, *ACTG1*, *TMC1*, and *POU4F3* genes (Table 2). Of them, three were novel missense mutations (p.G38D in *COCH*, p.E316K in *ACTG1*, and p.P164R in *POU4F3*), one was a novel in-frame indel mutation (c.2036-2038delAGG in *WFS1*), and two were known deafness-causing mutations that have been previously reported (p.R653C in *WFS1* and p.D572N in *TMC1*).

**Table 2:** **Mutations identified in 7 families**

**Family ID**	**Gene**	**DFN locus**	**Nucleotide change**	**Variation type**	**Amino acid change**	**Chromosome/exon**	**Mutation taster**	**PROVEAN** ^**a**^	**SIFT** ^**b**^	**Allele frequency in controls**	**Novel or HGMD**
**Dominant**											
**JSNY-021**	*WFS1*	DFNA6/14/38	c.2036_2038	In-frame indel	p.E680del	4/8	DC^c^	-	-	0.00	Novel
			delAGG								
**JSNY-027**	*COCH*	DFNA9	c. 113G > A	Missense	p.G38D	14/4	DC	−1.431	0.00	0.00	Novel
**JSNY-033**	*WFS1*	DFNA6/14/38	c.1957C > T	Missense	p.R653C	4/8	DC	−3.501	0.98	0.00	Awata *et al*. [14]
**JSNY-043**	*ACTG1*	DFNA20/26	c.946G > A	Missense	p.E316K	17/5	DC	−2.648	0.01	0.00	Novel
**JSNY-053**	*TMC1*	DFNA36	c.1714G > A	Missense	p.D572N	9/19	DC	−2.499	0.21	0.00	Kurima *et al*. [15]
**JSNY-056**	*POU4F3*	DFNA15	c.491 C > G	Missense	p.P164R	5/2	DC	−2.112	0.34	0.00	Novel
**Recessive**											
**JSNY-045**	*USH2A*	1q41	c.5051G > A	Missense	p.P1684L	1/25	DC	−4.567	0.00	0.00	Novel

^a^Negative and positive scores indicate deleterious and neutral, respectively, with cut-off score set at −1.3; ^b^Score ranges from 0 (deleterious) to 1 (neutral), with cut-off score set at 0.05. ^c^DC: Disease causing.

**Figure 2:**
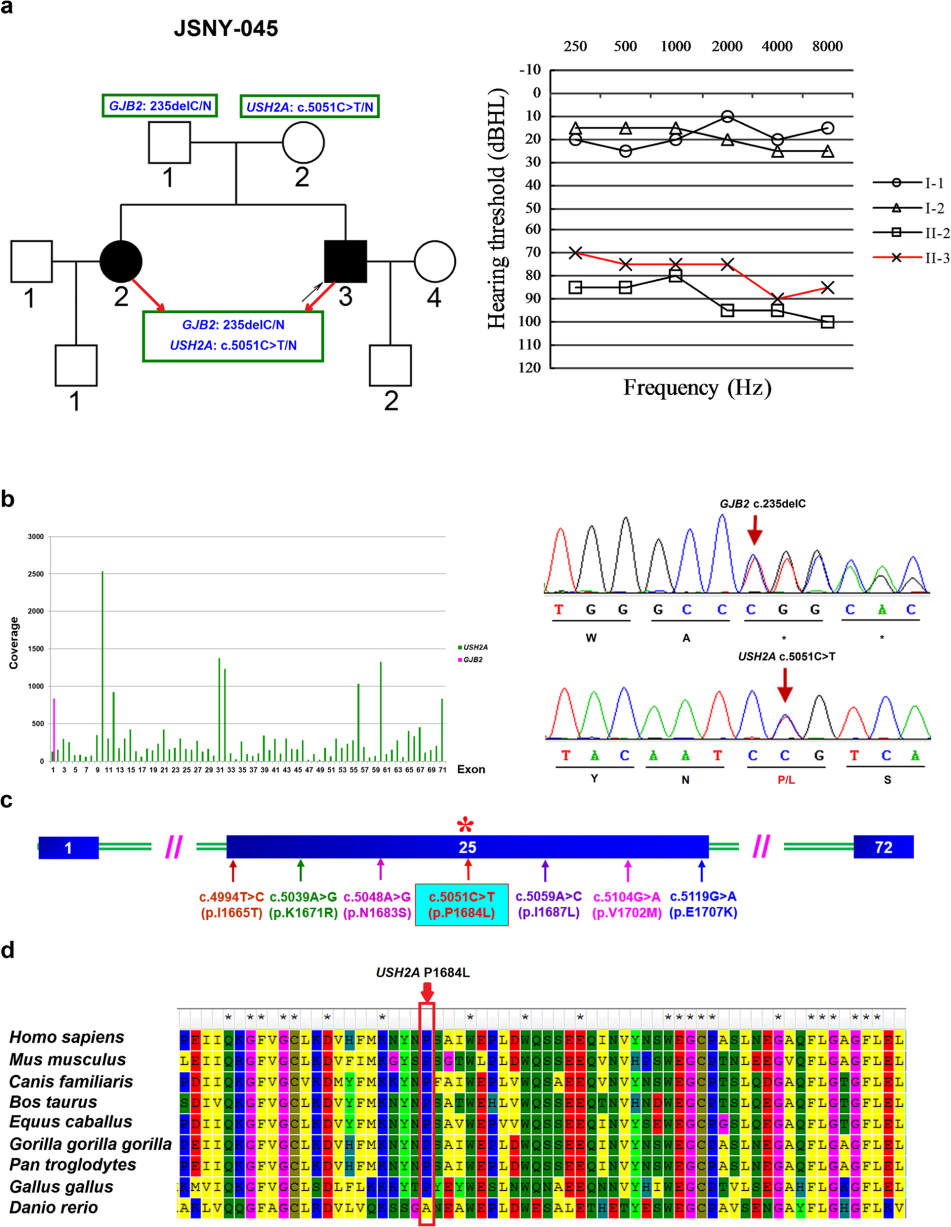
**DFNB in the affected family segregated with the** ***GJB2*** **c.235delC and** ***USH2A*** **c.5051C > T mutations. (a)** Family pedigree of JSNY-045 and audiograms of two deaf siblings and their parents. **(b)** The average coverage of each exon of the *GJB2* and *USH2A* genes in the proband (left) and sequencing electropherograms of the heterozygous *GJB2* c.235delC and *USH2A* c.5051C > T mutations (right). **(c)** A novel c.5051C > T variant and six previously reported mutations in *USH2A* exon 25. **(d)** Conservation analysis of the novel missense mutation. The *USH2A* p.P1684L mutation occurs at an evolutionarily conserved amino acid (in red box).

Another family possessed a candidate variant (JSNY-045) exhibiting autosomal recessive inheritance. Both siblings II-2 and II-3 suffered from severe sensorineural hearing loss that was congenital and non-progressive (Figure 2a). None of them reported eye problems. Complete ophthalmic examination showed negative findings (Table 3). During routine mutation detection, the proband (II-3) was found to be a carrier of the monoallelic *GJB2* c.235delC mutation. Subsequent targeted capture sequencing of known deafness genes confirmed the presence of this mutation as well as of a newly detected heterozygous *USH2A* gene mutation (c.5051C > T, p.P1684L) located in exon 25 (Figure 2c). We then used Sanger sequencing to screen the proband’s sibling and parents. The results showed that the sibling possessed the same genotype as the proband (Figure 2b), while the parents were each unaffected heterozygotes carrying a *GJB2* c.235delC monoallelic mutation (the father) or a *USH2A* c.5051C > T monoallelic mutation (the mother).

**Table 3:** **Clinical manifestation of JSNY-045 family members**

**Members**	**Gender**	**Age at enrollment (years)**	**Clinical testing**					
			**PTA** ^**a**^ **(dBHL)**	**Vestibular function**	**Night blindness**	**Visual fields**	**Photophobia**	**ERG** ^**b**^
II3	Male	28	78.75	Normal	No	Normal	No	Normal
II2	Female	30	88.75	Normal	No	Normal	No	Normal
I1	Male	55	18.75	Normal	No	Normal	No	Normal
I2	Female	53	18.75	Normal	No	Normal	No	Normal

^a^PTA: pure tone average of 0.5, 1, 2, and 4 kHz for the better ear; ^b^ERG: electroretinogram.

To predict the probable pathogenic effects of the above variants, we analyzed the evolutionary conservation and damaging effects of the amino acid substitutions. The results showed that all 7 of these variants were highly conserved among multiple vertebrate species (Figure 2d, Additional file 3: Figure S1). The results from the prediction analysis are shown in Table 2.

None of the candidate mutations was found in the 195 Chinese Han healthy controls.

## Discussion

In the present study, we confirmed the presence of seven non-synonymous variants in 7 of the 23 deafness families. Six families likely had causative mutations in DFNA genes [*WFS1* (n = 2), *ACTG1*, *POU4F3*, *COCH* and *TMC1*], and one showed two monoallelic and most likely causative mutations in the *GJB2* and *USH2A* genes, respectively. With the exception of the p.R653C mutation in *WFS1* and the p.D572N mutation in *TMC1*, the other five variants have not been previously reported to be associated with hereditary hearing loss. The causative genes detected in the remaining 16 families require further examination, presumably by linkage analysis and/or WES. It is probable that hearing loss in these families is due to mutations in unidentified deafness-related genes. Alternatively, pathogenic mutations might exist in those regions not covered in our sequencing analysis, including intronic regulatory sequences.

In the JSNY-021 family, a novel in-frame indel mutation caused by c.2036_2038delAGG (p.E680del) was detected in *WFS1*, which is responsible for DFNA6/14/38 hearing loss. This gene encodes wolframin, which is a membrane glycoprotein predominantly located in the endoplasmic reticulum (ER). This protein is essential for maintaining correct levels of Ca^2+^ and other charged particles necessary for hearing, and its lack of function induces apoptotic input signaling in the ER [16,17]. Although it remains unknown whether wolframin is expressed in the human cochlea, mutations in the *WFS1* gene, such as c.511G > A (p.D171N), c.2005 T > C (p.Y669H) and c.2590G > A (p.E864K), have been identified as frequent causes of autosomal dominant low-frequency hearing loss in different ethnicities [18-20]. The heterozygous 3-bp deletion (c.2036_2038delAGG) identified in the present study is expected to cause the loss of the E680 codon, which might affect the three-dimensional shape or properties of the wolframin protein and consequently interfere the normal function of the wolframin tetramer. Considering previous studies and the low-frequency NSHL phenotype found in the JSNY-021 family in this study, we hypothesize that this mutation likely has a pathogenic effect.

*ACTG1* encodes cytoskeletal actin gamma 1, which is known to be the building block of hair cell stereocilia. These stereocilia are constantly undergoing actin polymerization at their tips and depolymerization at their bases [21]. In auditory hair cells of the cochlea and intestinal epithelial cells, actin gamma 1 has a predominant and unique expression pattern. Mutations in the *ACTG1* gene have been mainly associated with autosomal dominant progressive sensorineural deafness 20/26 (DFNA20/26), and some have been linked to Baraitser-Winter syndrome, which is a rare autosomal recessive disorder characterized by developmental delay, facial dysmorphology, brain malformations, coloboma, and variable hearing loss [22-24]. To date, a total of 19 *ACTG1* mutations have been reported in patients with DFNA 20/26 and Baraitser-Winter syndrome, of which 12 have been identified in NSHL families (http://www.hgmd.org/, designed by P.D.Stenson HGMD®). In this study, we found the novel missense mutation p.E316K, which was caused by a c.946 G > A transition in *ACTG1*, in the JSNY-043 family. p.E316K is located in subdomain 3 of actin gamma 1, which is a highly conserved actin domain of ACTG1. Until now, all reported *ACTG1* missense mutations have been located in this domain.

The POU4F3 protein is a well-known transcription factor encoded by *POU4F3*. This protein belongs to the POU-domain class IV transcription factor family and plays an important role in the maturation, differentiation and survival of hair cells [25]. Mutations in the *POU4F3* gene have been described in patients with nonsyndromic sensorineural deafness autosomal dominant type 15 (DFNA15). To date, more than five different mutation types, including the deletion of the entire *POU4F3* gene sequence, have been reported worldwide in different ethnic groups [26,27], but none have been reported in the Asian population. In the present study, the *POU4F3* p.P164R missense mutation caused by c.491C > G was identified as a novel mutation in the JSNY-056 Chinese family.

The *COCH* gene encodes a 550-amino acid protein with multiple domains, including a signal peptide (SP), an LCCL module and two von Willebrand factor A (vWFA) domains [28]. The COCH protein, cochlin, is abundantly expressed in the cochlea and vestibular system of the inner ear. Mutations in the *COCH* gene lead to autosomal dominant nonsyndromic sensorineural deafness 9 (DFNA9), which has been clinically characterized by progressive late-onset hearing loss with or without vestibular dysfunction [29]. Presently, 18 *COCH* mutations have been identified in DFNA9 families (http://www.hgmd.org/, designed by P.D.Stenson HGMD®), most of which are located in the LCCL region. Here, we identified a novel missense mutation (c.113G > A, p.G38D) in exon 3 of the *COCH* gene, which is the LCCL domain of cochlin. Mutations in this domain are expected to cause misfolding and protein aggregation in a dominant-negative manner, leading to cytotoxicity of the inner ear fibrocytes [30]. The clinical features associated with this novel mutation in the JSNY-027 family lends support to the pathogenic nature of the p.G38D variant.

In the present study, we also identified one novel mutation of *USH2A* c.5051C > A (p.P1684L) in the JSNY-045 family. *USH2* mutations result in an autosomal recessive disorder characterized by retinitis pigmentosa and mild-to-moderate sensorineural hearing loss, and the *USH2A* gene is most commonly mutated. This gene is located at 1q41 and encodes a protein with a predicted size of 171.5 kDa [31,32]. To date, more than 120 different disease-causing mutations have been reported in the *USH2A* gene, which are widely distributed over the coding regions of all 72 exons. Interestingly, the novel p.P1684L mutation, which is located in exon 25 of the *USH2A* gene, was identified together with the *GJB2* c.235delC mutation in two deaf siblings of the JSNY-045 family, while their hearing parents each carried only one monogenic recessive mutation. All subjects exhibited normal vestibular and visual functions. We therefore deduced that the *USH2A* monogenic recessive mutation failed to cause the Usher phenotype but may have contributed to the pathogenesis of the *GJB2* c.235delC mutation, resulting in phenotypic hearing loss in the two patients. In fact, digenic inheritance of nonsyndromic deafness caused by mutations in the *GJB2* gene and other connexin genes, such as *GJB3*, *GJB6*, *GJB4*, or *GJA1*, have been previously reported in several deaf patients [33-35]. In addition, a Japanese family with comorbid *GJB2* and *WFS1* mutations was also described in 2012. In this family, one individual with mutations in both *GJB2* and *WFS1* presented with a *GJB2* phenotype [36]. Our results together with previous findings suggest that monoallelic *GJB2* mutations may contribute to NSHL as a result of their co-inheritance with other deafness-causing genes. However, the pathogenic mechanisms underlying the cooperation of these genes with *GJB2* require further research.

## Conclusions

Using targeted genomic capture and MPS, we have successfully identified causative gene mutations in six families with autosomal dominant NSHL, and in patients from a family with recessive DFNB, we detected two monoallelic mutations in the *GJB2* and *USH2A* genes, respectively. The *USH2A* gene may have cooperated with *GJB2* to cause the NSHL phenotype in this Chinese family. Our results suggest that targeted genomic capture combined with MPS can serve as a useful technique in the etiological diagnosis of sensorineural hearing loss.

## Additional files

Additional file 1: Table S1. Target genes and regions of the DeafPanel. Additional file 2: Table S2. PCR primers surrounding the suspected variants of 7 genes. Additional file 3: Figure S1. Sanger sequencing and conservation analysis of gene mutations identified in 6 autosomal dominant families. **(a)** *WFS1* c.2036_2038delAGG (p.E680del) in-frame indel mutation in the JSNY-021 family. **(b)** *COCH* c.113G > A (p.G38D) missense mutation in the JSNY-027 family. **(c)** *WFS1* c.1957C > T (p.R653C) missense mutation in the JSNY-033 family. **(d)** *ACTG1* c.946G > A (p.E316K) missense mutation in the JSNY-043 family. **(e)** *TMC1* c.1714G > A (p.D572N) missense mutation in the JSNY-053 family. **(f)** *POU4F3* c.491C > G (p.P164R) missense mutation in the JSNY-056 family.
