# Supplementary material for: Targeted genomic capture and massively parallel sequencing to identify novel variants causing Chinese hereditary hearing loss
Source: J Transl Med. 2014 Nov 12;12:311. doi: 10.1186/s12967-014-0311-1 (PMC4234825; doi:10.1186/s12967-014-0311-1)
Supplement: Additional file 1: Table S1. — Target genes and regions of the DeafPanel. [file 12967_2014_311_MOESM1_ESM.doc]

**Additional file 1: Table S1 Target genes and r**egions of the DeafPanel

| **Deafness-related nuclear genes** | | | | | | | |
| --- | --- | --- | --- | --- | --- | --- | --- |
| *ACTG1* | *EYA4* | *MYO6* | *STRC* | *ECM1* | *KRT9* | *CCDC50* | *GJB2* |
| *MYO7A* | *TECTA* | *PRPS1* | *TCOF1* | *CDH23* | *GJB3* | *NF2* | *TIMM8A* |
| *FGFR3* | *GATA3* | *CLDN14* | *GJB6* | *OTOA* | *TMC1* | *FGF3* | *HMX1* |
| *CLRN1* | *GPR98* | *OTOF* | *TMIE* | *FOXI1* | *PABPN1* | *COCH* | *GRHL2* |
| *PAX3* | *TMPRSS3* | *FGFR1* | *GRXCR1* | *COL11A2* | *HARS* | *PCDH15* | *TRIOBP* |
| *PROKR2* | *HGF* | *CRYM* | *KCNQ4* | *PJVK* | *TRMU* | *PROK2* | *MIR96* |
| *DFNA5* | *LHFPL5* | *POU3F4* | *USH1C* | *CHD7* | *DFNB59* | *DFNB31* | *MARVELD2* |
| *POU4F3* | *USH1G* | *FGF8* | *LRTOMT* | *DIAPH1* | *MITF* | *RDX* | *USH2A* |
| *IL13* | *LOXHD1* | *DSPP* | *MYH14* | *SANS* | *VLGR1* | *LAMA3* | *TPRN* |
| *EDN3* | *MYH9* | *SLC26A4* | *WFS1* | *HOXA2* | *KCNQ1* | *EDNRB* | *MYO15* |
| *SLC26A5* | *WHRN* | *SIX1* | *KCNE1* | *ESPN* | *MYO1A* | *SNAI2* | *SALL4* |
| *SLC17A8* | *SALL1* | *ESRRB* | *MYO3A* | *SOX10* | *FLNA* | *MYO15A* | *RPGR* |
| *FREM1* | *SIX5* | *EYA1* | *ALX3* | *SEC23A* |  |  |  |
| **Deafness-related mitochondrial DNA** | | | | | | | |
| *RNR1* | | | | chrM:601-1680 | | | |
| *TS1* | | | | chrM:7381-7591 | | | |
| *CO1* | | | | chrM:5881-7500 | | | |
| **Deafness-related microRNAs** | | | | | | | |
| *miR-96* | | | | chr7: 129414532-129414609 | | | |
| *miR-182* | | | | chr7: 129410223-129410332 | | | |
| *miR-183* | | | | chr7: 129414745-129414854 | | | |
