# Supplementary material for: Targeted genomic capture and massively parallel sequencing to identify novel variants causing Chinese hereditary hearing loss
Source: J Transl Med. 2014 Nov 12;12:311. doi: 10.1186/s12967-014-0311-1 (PMC4234825; doi:10.1186/s12967-014-0311-1)
Supplement: Additional file 2: Table S2. — PCR primers surrounding the suspected variants of 7 genes. [file 12967_2014_311_MOESM2_ESM.doc]

**Additional file 2: Table S2 PCR primers surrounding the suspected variants of 7 genes**

| **Primers** | **Sequence(5′to 3′)** | **Tm(**°C**)** | **Size (bp)** |
| --- | --- | --- | --- |
| *WFS1* | FW:5'-TCGTGCTGTTCTGCTGGTTCT-3'  RV:5'-TGCCCACGGTAATCTCAAACT-3' | 58.5 | 414 |
| *COCH* | FW: 5’-CCCAGAACTTTCACATTAGAG-3’  RV:5’-GGAGAACCGTGAAAATATATC-3’ | 59.5 | 536 |
| *WFS1* | FW:5’-GTCTGTAGTGTGCCCCTGCT-3’  RV:5’-GATGGTGCTGAACTCGATGA-3’ | 57.7 | 678 |
| *ACTG1* | FW:5’-CGGTGACACAGCATCACTAAG-3’  RV:5’-ACGGCTTCAGCTCACAGAG-3’ | 55.0 | 279 |
| *TMC1* | FW: 5’-CCTGTCTTGCTGTACTTTCAT-3’  RV: 5’-TATGGAGTTAGACACCGATTG-3’ | 57.5 | 422 |
| *POU4F3* | FW:5’-GCACATCTCGCCCACGCTGA-3’  RV:5’-CCACGCCGGGGATCTTGAGA-3’ | 59.0 | 299 |
| *USH2A* | FW: 5’-CGGCAGATATGATGGGTTTG-3’  RV: 5’-GGAATGTAATGAAGCGGGAC-3’ | 57.2 | 375 |

FW: forward primer; RV: reverse primer
