# Supplementary figures and images for: Targeted genomic capture and massively parallel sequencing to identify novel variants causing Chinese hereditary hearing loss
Source: J Transl Med. 2014 Nov 12;12:311. doi: 10.1186/s12967-014-0311-1 (PMC4234825; doi:10.1186/s12967-014-0311-1)

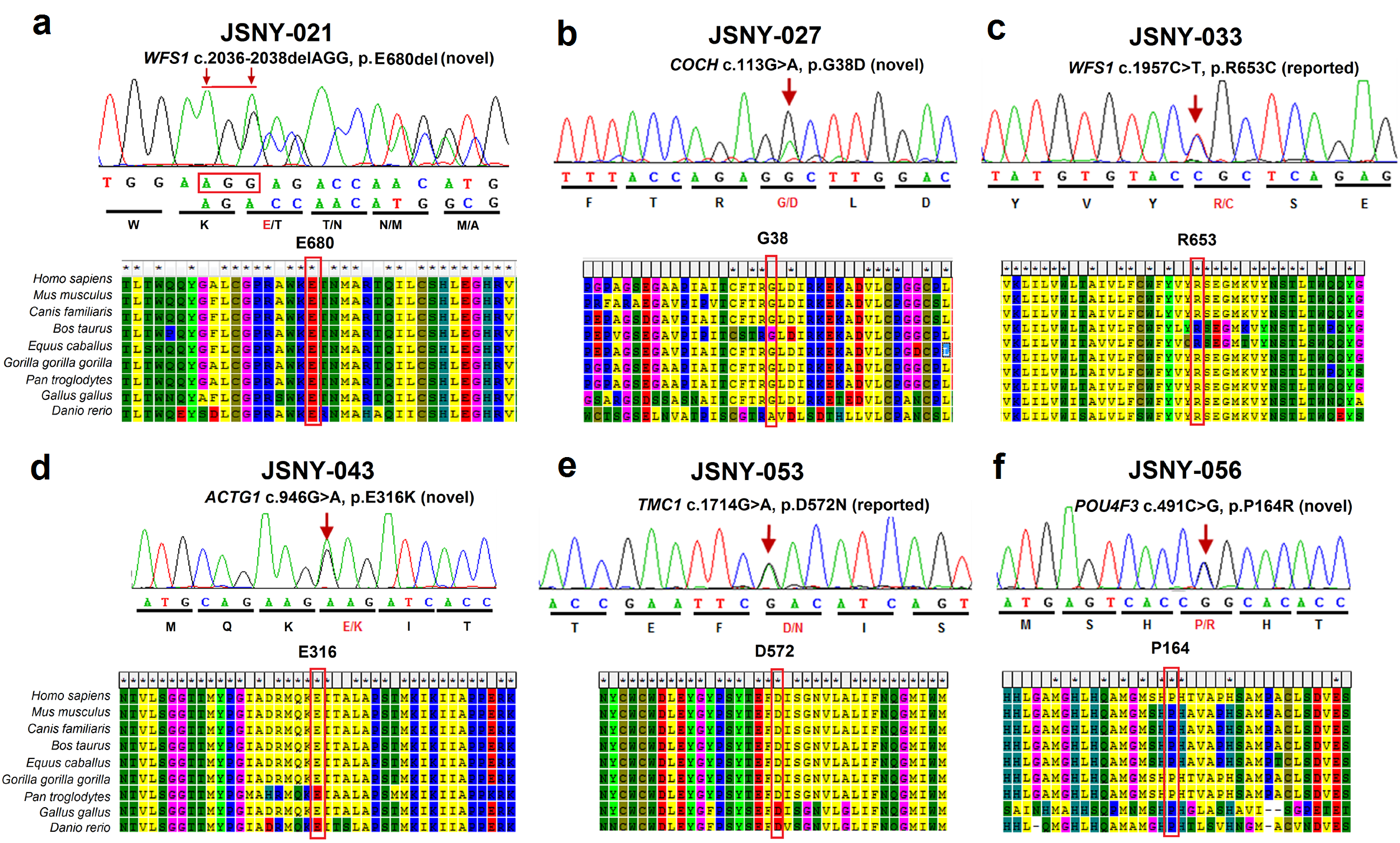

Supplement: Additional file 3: Figure S1. — Sanger sequencing and conservation analysis of gene mutations identified in 6 autosomal dominant families. (a) WFS1 c.2036_2038delAGG (p.E680del) in-frame indel mutation in the JSNY-021 family. (b) COCH c.113G > A (p.G38D) missense mutation in the JSNY-027 family. (c) WFS1 c.1957C > T (p.R653C) missense mutation in the JSNY-033 family. (d) ACTG1 c.946G > A (p.E316K) missense mutation in the JSNY-043 family. (e) TMC1 c.1714G > A (p.D572N) missense mutation in the JSNY-053 family. (f) POU4F3 c.491C > G (p.P164R) missense mutation in the JSNY-056 family. [file 12967_2014_311_MOESM3_ESM.tiff]
